# The utility of the systemic immune inflammation and systemic inflammation response indices in suspected intermediate-risk pulmonary embolism

**DOI:** 10.3325/cmj.2024.65.13

**Published:** 2024-02

**Authors:** Muhammed Fuad Uslu, Mustafa Yilmaz, Metin Ateşçelik, Feti Ahmet Atilgan

**Affiliations:** 1 Department of Internal Medicine, Elazig Fethi Sekin City Hospital, Elazig, Turkey; 2 Department of Emergency Medicine, Firat University of Medicine, Elazig, Turkey; 3 Department of Emergency Medicine, Elazig Fethi Sekin City Hospital, Elazig, Turkey

## Abstract

**Aim:**

To evaluate the utility of the systemic immune inflammation index (SII) and systemic inflammation response index (SIRI) in diagnosing pulmonary embolism (PE) in emergency medicine.

**Methods:**

We retrospectively reviewed the data of patients who presented to the emergency department and underwent contrast-enhanced computed tomography pulmonary angiography for suspected PE between January 1 and December 31, 2021. In 81/168 patients, the diagnosis of PE was confirmed and in 87/168 it was rejected. The data were analyzed with receiver operating characteristic analysis and binary logistic regression analysis.

**Results:**

Patients with PE had a higher white blood cell count (*P* < 0.001), neutrophils (*P* = 0.002), monocytes (*P* = 0.013), neutrophil/lymphocyte ratio (*P* < 0.001), SII (*P* < 0.001), and SIRI (*P* < 0.001), and a lower lymphocyte count (*P* = 0.002). The SII had a sensitivity of 75.31% and a specificity of 71.26%, while the SIRI had a sensitivity of 82.72% and a specificity of 68.97%. Binary logistic regression analysis showed that the Wells score, D-dimer level, and SII independently influenced the diagnosis of PE.

**Conclusion:**

The SII and SIRI may be used to support the diagnosis of PE in the emergency department.

Pulmonary embolism (PE) is a prevalent cause of mortality globally. Its underlying mechanisms are intricately tied to increased blood clotting tendencies, damage to the endothelial lining, and the presence of inflammation (1). Well-known predictors of all-cause mortality in pulmonary embolism are elevated neutrophil/lymphocyte ratio (NLR) and platelet-to-lymphocyte ratio (PLO) (2,3). Recently, two novel markers have emerged – the systemic immune-inflammation index (SII) and systemic immune response index (SIRI). Due to their accessibility, affordability, practicality, and non-invasive nature, these two indices offer potential advantages in the simultaneous assessment of various parameters such as platelet count, neutrophil count, lymphocyte count, and monocyte count. The SII (platelet count × neutrophil count/lymphocyte count) and the SIRI (neutrophil count × monocyte count/lymphocyte count) have been proven as valuable predictors of adverse clinical outcomes in patients with cancer and inflammatory conditions (4,5). While recent research has proposed the applicability of the SII and SIRI as markers for massive/submassive PE (6), there has been a scarcity of studies examining their effectiveness in the diagnosis of PE in the emergency department setting. The aim of this study was to assess the usefulness of the SII and SIRI in differentiating patients with and without PE who present to the emergency department with high D-dimer levels and who undergo computed tomography pulmonary angiography (CTPA) for suspected intermediate-risk PE.

## PATIENTS AND METHODS

**Figure 1:**
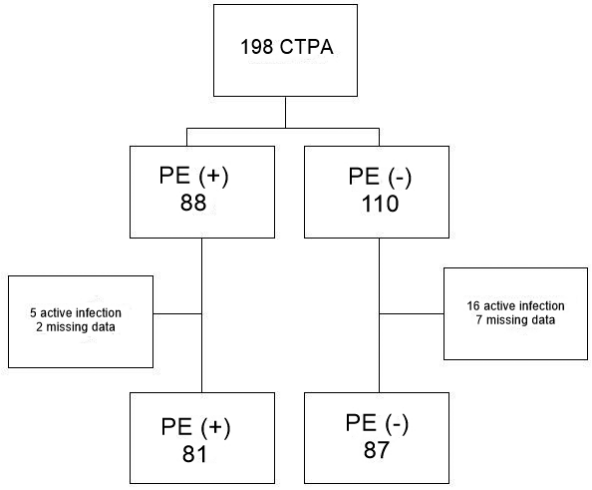
The study flowchart. PE – pulmonary embolism; CTPA – computed tomography pulmonary angiography.

This retrospective study encompassed individuals aged 18 years or older who were admitted to the department of emergency medicine at Firat University between January 1 and December 31, 2021 for respiratory complaints. They underwent CTPA due to elevated age-adjusted D-dimer levels. Based on the results of the CTPA, the diagnosis of PE was either confirmed or rejected. Patients were excluded from the study if they had an active infection (Figure 1). The inclusion criteria were having had D-dimer measured and having undergone CTPA. Patients who were considered as low-risk for developing PE did not undergo CTPA and were therefore not included in the study. The patients were categorized into the PE group and the control group. The study was approved by the Ethics Committee of the Firat University of Medicine.

### Laboratory measurements

Serum biochemical parameters were measured with the Advia 2400 Chemistry System (Siemens Diagnostics, Tarrytown, NY, USA); hematological parameters with the Advia 2120i (Siemens, Munich, Germany), and plasma D-dimer levels with Sysmex CS5100 (Sysmex, Kobe, Japan).

### Statistical analysis

The normality of distribution of continuous variables was tested with a Shapiro-Wilk test. The Mann-Whitney test was used to compare continuous variables between two groups. The χ^2^ test (cross tabulation) and Fisher exact test were used to compare categorical data. Furthermore, Spearman correlation analysis and receiver-operating characteristic (ROC) curve analysis were conducted. Statistical analysis was performed with SPSS 21.0 (IBM Corp., Armonk, NY, USA). *P* < 0.05 was considered statistically significant.

## RESULTS

The study encompassed 168 patients who presented to the emergency department with symptoms suggestive of PE and subsequently underwent CTPA. In 81 of these patients, the diagnosis of PE was confirmed, and in 87 it was rejected. There was no significant difference between the groups in the distribution of male and female patients (51.8% women in the PE group and 63.2 in the control group; *P* = 0.091). The average age of patients diagnosed with PE was significantly greater than that of control participants (*P* = 0.033). Regarding risk factors, participants diagnosed with PE exhibited a significantly higher likelihood of experiencing tachycardia (*P* = 0.035) and hemoptysis (*P* = 0.005). There was no difference between the groups in terms of immobilization (*P* = 0.266), history of PE (*P* = 0.061), and cancer (*P* = 0.182). Furthermore, patients with PE showed significantly elevated Wells scores (*P* < 0.001), D-dimer levels (*P* < 0.001), and CRP levels (*P* = 0.001). They also had significantly higher WBC, neutrophils, monocytes, NLR, SII, and SIRI and lower lymphocyte count (Table 1). There was no difference in NLR, SII, and SIRI levels depending on the presence or absence of PE risk factors (Table 2).

**Table 1:** Sociodemographic characteristics and laboratory findings in controls and participants diagnosed with pulmonary embolism (PE)

	Control	PE	p
N (female/male)	87 (55/32)	81 (42/39)	0.091*
Age, mean±SD (years)	65.9 ± 17.05	71.4 ± 16.30	0.033^†^
History of deep venous thrombosis, n (%)	9 (10.3)	11 (13.6)	0.341*
Tachycardia, n (%)	26 (29.9)	37 (45.7)	0.035*
Cancer, n (%)	7 (8.0)	11 (13.6)	0.182*
Immobilization, n (%)	20 (23.0)	23 (28.4)	0.266*
History of PE, n (%)	6 (6.9)	13 (16.0)	0.061*
Hemoptysis, n (%)	0	7 (8.6)	0.005^‡^
Wells score, median (IQR)	4.50 (3.00-5.50)	5.50 (4.50-6.00)	<0.001^§^
D-dimer (µg/mL), median (IQR)	1.32 (0.95-2.33)	4.67 (2.30-9.69)	<0.001^§^
C-reactive protein (mg/dL), median (IQR)	13.80 (4.94-31.75)	28.20 (12.23-62.55)	0.001^§^
White blood cell (10^3^/mm^3^), median (IQR)	7.89 (6.48-9.41)	9.19 (7.45-12.10)	<0.001^§^
Lymphocyte (10^3^/μL), median (IQR)	2.05 (1.55-3.15)	1.72 (1.27-2.29)	0.002^§^
Neutrophil (10^3^/μL), median (IQR)	4.65 (3.57-5.64)	6.89 (4.77-8.85)	<0.001^§^
Monocytes (10^3^/μL), median (IQR)	0.62 (0.47-0.76)	0.71 (0.54-0.99)	0.013^§^
Hemoglobin (mg/dL), median (IQR)	13.10 (11.80-14.30)	13.20 (11.50-14.55)	0.951^§^
Hematocrit (%), median (IQR)	40.30 (37.40-44.60)	40.80 (36.20-44.95)	0.792^§^
Platelet (10^3^/mm^3^), median (IQR)	234.00 (203.00-291.00)	253.00 (202.00-316.50)	0.223^§^
Neutrophil/lymphocyte ratio, median (IQR)	2.04 (1.49-3.19)	4.02 (2.81-5.95)	<0.001^§^
Systemic immune-inflammation index, median (IQR)	531.16 (344.70-756.99)	1003.92 (650.27 -1533.98)	<0.001^§^
Systemic inflammation response index, median (IQR)	1.26 (0.80-1.91)	2.48 (1.77-4.94)	<0.001^§^

*χ^2^ test.

†t-test.

‡Fisher exact test.

§Mann-Whitney U test.

**Table 2:** The values of neutrophil/lymphocyte ratio (NLR), the systemic immune inflammation (SII), and the systemic inflammation response index (SIRI) based on the presence or absence of risk factors for pulmonary embolism

Risk factor		NLR	SII	SIRI
		Median (IQR)	Median (IQR)	Median (IQR)
DVT	present (n = 11)	3.53 (2.48-5.82)	797.90 (573.94-1158.78)	2.12 (1.77-5.17)
	absent (n = 70)	4.11 (2.84 -6.08)	1027.06 (708.10-1598.25)	2.55 (1.76-4.89)
	p^†^	0.649	0.276	0.684
Tachycardia	present (n = 37)	4.11 (2.84-5.48)	844.95 (529.29-1519.90)	2.36 (1.80-4.51)
	absent (n = 44)	3.85 (2.67-6.09)	1014.35 (720.32-1569.28)	2.62 (1.71-6.05)
	p^†^	0.989	0.609	0.824
Cancer	present (n = 11)	6.27 (3.17-11.32)	1434.89 (998.57-2112.69)	4.37 (2.25-6.34)
	absent (n = 70)	3.65 (2.75-5.48)	910.79 (586.14-1501.55)	2.36 (1.76-4.48)
	p^†^	0.066	0.080	0.110
Immobilization	present (n = 23)	4.02 (2.56-5.66)	1024.78 (527.30-1684.74)	2.48 (1.71-4.43)
	absent (n = 58)	4.03 (2.84-6.09)	1001.25 (713.71-1517.93)	2.47 (1.80-5.14)
	p^†^	0.652	0.810	0.757
History of PE	present (n = 13)	3.61 (2.71-4.28)	1033.06 (655.17-1694.10)	1.97 (1.73-3.99)
	absent (n = 68)	4.20 (2.83-6.09)	1001.25 (621.51-1521.86)	2.55 (1.80-5.11)
	p^†^	0.448	0.837	0.354
Hemoptysis	present (n = 7)	3.35 (1.67 -4.65)	976.62 (502.81-1024.78)	2.25 (0.85-4.59)
	absent (n = 74)	4.07 (2.81-6.09)	1031.20 (708.10-1598.25)	2.51 (1.77-5.06)
	p^†^	0.264	0.220	0.501

*DVT – deep vein thrombosis.

^†^Mann-Whitney U test.

ROC analysis was conducted to assess the predictive value of the SII, SIRI, NLR, D-dimer, and the Wells score in distinguishing patients with PE from the control group. The SII had a sensitivity of 75.31% and a specificity of 71.26%, while the SIRI had a sensitivity of 82.72% and a specificity of 68.97% (Figure 1, Table 3).

**Table 3:** Receiver-operating characteristic analysis results showing the performance of different tests for predicting pulmonary embolism*

	Cut-off	Area under the curve	95% confidence interval	Sensitivity	Specificity	Positive predictive value	Negative predictive value	p
D-dimer	>2.35	0.810	0.742-0.866	75.31	77.01	75.3	77.0	<0.001
Wells score	>4.5	0.697	0.622-0.766	61.73	72.41	67.6	67.0	<0.001
SII	>705.6	0.797	0.728-0.855	75.31	71.26	70.9	75.6	<0.001
SIRI	>1.59	0.791	0.721-0.850	82.72	68.97	71.3	70.3	<0.001
NLR	>2.39	0.803	0.735-0.861	85.19	63.22	68.3	82.1	<0.001

*Abbreviations: NLR – neutrophil/lymphocyte ratio; SII – systemic immune inflammation index; SIRI – systemic inflammation response index.

**Figure 2:**
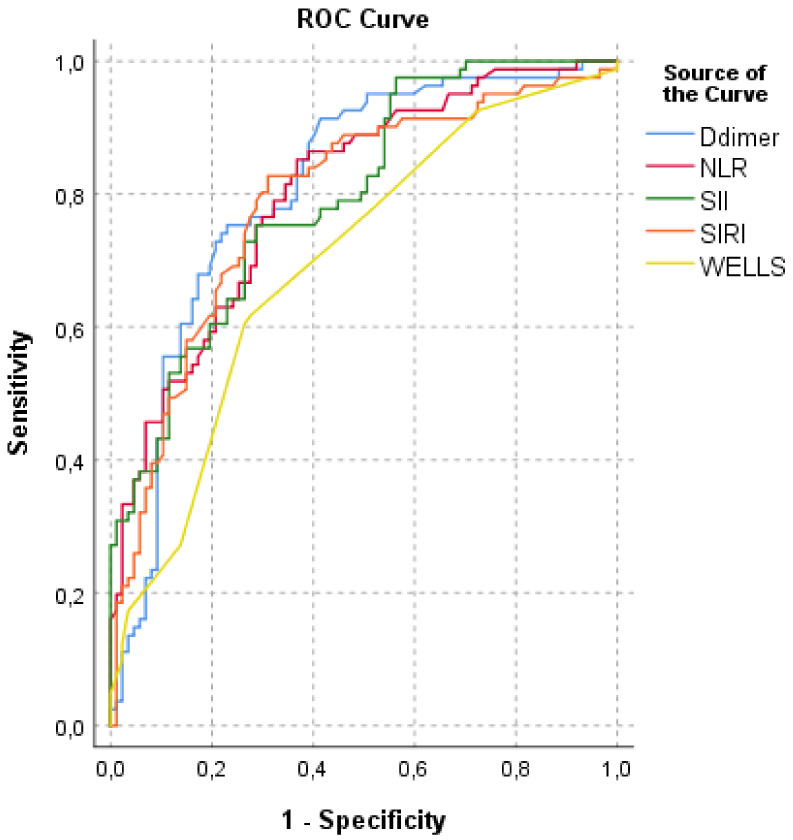
Receiver-operating characteristic (ROC) analysis showing the predictive value of different indicators for pulmonary embolism. NLR – neutrophil/lymphocyte ratio; SII – systemic immune inflammation index; SIRI – systemic inflammation response index.

ROC analysis showed that the cut-off values obtained with 100% sensitivity for D-dimer (cut-off <0.69) excluded PE diagnosis in 5 patients (5.7%); those obtained for NLR (cut-off <1.12) excluded PE diagnosis in 6 patients (6.9%); and those obtained for the SII (cut-off <356.15) excluded PE diagnosis in 25 patients (28.7%). However, the Wells score (cut-off <0.5) and the SIRI (cut-off <0.15), at the cut-off values obtained with 100% sensitivity, did not safely exclude PE diagnosis in any of the patients (Figure 2).

When the SII was higher than 705.6, the risk of PE was approximately 7 times higher than when it was lower than 705.6. When the SIRI was higher than 1.59, the risk of PE was approximately 10 times greater than when it was lower than 1.59 (Table 4).

**Table 4:** The contingency table of indices in the diagnosis of pulmonary embolism*

	Pulmonary embolism				
Variable, n (%)	yes	no	p	Odds ratio	95% confidence interval
Wells score					
>4.5	50 (61.7)	24 (27.6)	<0.001	4.234	2.211-8.106
≤4.5	31 (38.3)	63 (72.4)			
D-dimer					
>2.35	61 (75.3)	20 (23.0)	<0.001	10.218	5.022-20.788
≤2.35	20 (24.7)	67 (77.0)			
SII					
>705.6	61 (75.3)	25 (28.7)	<0.001	7.564	3.809-15.021
≤705.6	20 (24.7)	62 (71.3)			
SIRI					
>1.59	67 (82.7)	27 (31.0)	<0.001	10.635	5.107-22.146
≤1.59	14 (17.3)	60 (69.0)			
NLR					
>2.39	69 (58.2)	32 (36.8)	<0.001	9.883	4.658-20.966
≤2.39	12 (14.8)	55 (63.2)			

*Abbreviations: NLR – neutrophil/lymphocyte ratio; SII – systemic immune inflammation index; SIRI - systemic inflammation response index.

The Wells score, D-dimer level, and the SII independently influenced PE diagnosis (Table 5).

**Table 5:** Factors influencing the diagnosis of pulmonary embolism*

	Pulmonary embolism		
	Exp(B)	95% confidence interval	p
Constant	0.004		0.000
Age	0.990	0.966-1.016	0.459
Sex (female)	1.002	0.443-2.265	0.996
Wells score	1.907	1.345-2.702	<0.001
D-dimer	1.073	1.012-1.138	0.018
Neutrophil/lymphocyte ratio	1.283	0.777-2.119	0.329
Systemic immune inflammation index	1.002	1.000-1.004	0.020
Systemic inflammation response index	1.016	0.901-1.147	0.791

*R^2^ (Cox-Snell) = 0.388; R^2^ (Nagelkerke) = 0.517; *P* < 0.001.

## DISCUSSION

In our study, patients with PE had significantly higher NLR, SII, and SIRI than the control group. Additionally, the SII had a sensitivity of 75.31% and a specificity of 71.26%, while the SIRI had a sensitivity of 82.72% and a specificity of 68.97% in differentiating between PE patients and controls.

Major factors leading to the development of PE are hypercoagulability, endothelial damage, and inflammation. PE occurs when inflammation within the vessel wall triggers the formation of a thrombus in an undamaged vessel, as a result of the concurrent activation of both inflammation and coagulation (7). Numerous elements of the immune system, including cytokines, diverse leukocyte varieties, and chemokines, participate in the fundamental inflammatory mechanisms associated with thromboembolism (8). Inflammatory markers have been used in the diagnosis, prognosis prediction, and evaluation of mortality in PE patients (2,9).

The SII and SIRI have been recently established as markers for various types of cancer and inflammatory conditions (4,10). The SII and SIRI are also affected by vascular diseases. Higher SII and SIRI indices were linked to an increased risk of ischemic stroke severity, a finding that suggests the potential utility of the SII in predicting unfavorable clinical outcomes following an acute ischemic stroke (11). The same study also proposed that, compared with NLR and PLR, the SII and SIRI indices better reflected the comprehensive status of the immune system (11). The SII may function as a valuable indicator in clarifying the interplay between thrombocytosis, inflammation, and immunity associated with the onset of cerebrovascular diseases among middle-aged and elderly patients (10). The SII, which is composed of three hematologic parameters of inflammation (neutrophils, lymphocytes, and platelets), has been proposed as a prognostic marker for not only malignant diseases but also for coronary artery disease (CAD), whose development is affected by endothelial damage and inflammation. In patients with CAD, elevated SII scores were independently linked to coronary artery damage, an elevated risk of cardiac fatality due to heart failure, nonfatal myocardial infarction, nonfatal stroke, and hospitalization for heart failure following coronary intervention (12). Furthermore, the SII demonstrated superior predictive capability for major cardiovascular events compared with conventional risk factors in patients with CAD. Furthermore, elevated SII levels were found to correlate with the occurrence of massive acute PE (6). The same authors also observed that the SII may serve as an autonomous predictor of increased disease severity in patients with acute PE (6). They determined the optimal SII cut-off value to be >1161, with 91% sensitivity and 90% specificity. In our research, NLR exhibited a sensitivity of 85.19% and a specificity of 63.22% for diagnosing intermediate-risk PE. In the case of the SII, which is obtained by multiplying NLR by platelet count, sensitivity decreased to 75.31% but specificity increased to 71.26%.

Furthermore, an increased SII has been documented as an independent indicator of carotid intima-media thickness in hypertensive patients. This condition, along with CAD, leads to adverse cardiovascular outcomes, primarily due to endothelial dysfunction, atherosclerosis, and inflammatory processes (13,14).

NLR is a potentially useful indicator of critical stenosis and may be associated with the severity and characteristics of coronary atherosclerotic disease plaques (15,16). Many studies confirmed the utility of hematological factors such as NLR and PLR for diagnosing, prognosing, and assessing mortality in individuals with PE (2,3). Nevertheless, considering that the SII encompasses peripheral lymphocytes, neutrophils, and platelets, while the SIRI incorporates neutrophil count, monocyte count, and lymphocyte count, both of these metrics could serve as valuable indicators reflecting the pathways of thrombus formation, inflammatory responses, and adaptive immune reactions.

The SII and SIRI indices were found to be positively correlated with D-dimer levels. This study also revealed that NLR, SII, or SIRI values did not differ significantly in the presence or absence of cancer, DVT, or a history of PE.

The limitations of our study include its single-center and retrospective design. Additionally, we did not have access to the information on patients’ routine medication usage. Another limitation is the dependence of the diagnostic algorithm on the D-dimer test.

To summarize, the Wells score, D-dimer level, and SII independently influenced the diagnosis of PE. The SII and SIRI were higher in patients with PE. These indexes represent cost-effective and readily available indicators that can assist in reinforcing the diagnosis of PE in the emergency department.
